# SecYEG-mediated translocation in a model synthetic cell

**DOI:** 10.1093/synbio/ysae007

**Published:** 2024-05-10

**Authors:** Ludo L J Schoenmakers, Max J den Uijl, Jelle L Postma, Tim A P van den Akker, Wilhelm T S Huck, Arnold J M Driessen

**Affiliations:** Physical-Organic Chemistry, Institute for Molecules and Materials, Radboud University, Nijmegen 6525AJ, The Netherlands; Groningen Biomolecular Sciences and Biotechnology, Molecular Biotechnology, University of Groningen, Groningen 9747 AG, The Netherlands; General Instrumentation, Radboud University, Nijmegen 6525 AJ, The Netherlands; Groningen Biomolecular Sciences and Biotechnology, Molecular Biotechnology, University of Groningen, Groningen 9747 AG, The Netherlands; Physical-Organic Chemistry, Institute for Molecules and Materials, Radboud University, Nijmegen 6525AJ, The Netherlands; Groningen Biomolecular Sciences and Biotechnology, Molecular Biotechnology, University of Groningen, Groningen 9747 AG, The Netherlands

**Keywords:** synthetic cell, Sec translocon, translocation, liposomes, giant unilamellar vesicles

## Abstract

Giant unilamellar vesicles (GUVs) provide a powerful model compartment for synthetic cells. However, a key challenge is the incorporation of membrane proteins that allow for transport, energy transduction, compartment growth and division. Here, we have successfully incorporated the membrane protein complex SecYEG—the key bacterial translocase that is essential for the incorporation of newly synthesized membrane proteins—in GUVs. Our method consists of fusion of small unilamellar vesicles containing reconstituted SecYEG into GUVs, thereby forming SecGUVs. These are suitable for large-scale experiments while maintaining a high protein:lipid ratio. We demonstrate that incorporation of SecYEG into GUVs does not inhibit its translocation efficiency. Robust membrane protein functionalized proteo-GUVs are promising and flexible compartments for use in the formation and growth of synthetic cells.

## Graphical Abstract

**Figure:**
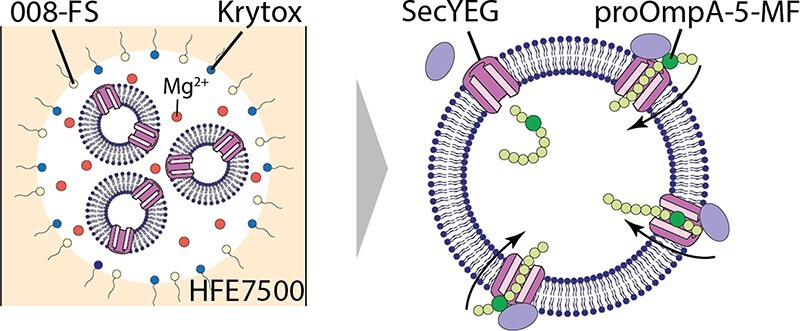

## 1. Introduction

One of the major challenges in synthetic biology is the bottom-up construction of a synthetic cell capable of autonomous growth and division. To accomplish this, it will be essential to provide control over a variety of cellular processes present in all forms of life, such as membrane biogenesis and membrane transport (1–3). Without the continuous insertion of newly synthesized membrane proteins, a synthetic cell quickly runs out of the energy and nutrients required to keep its complex internal chemistry in an out-of-equilibrium state. Furthermore, many crucial cellular processes such as phospholipid biosynthesis (1, 4–6) and cell division (7–9) are dependent on the activity of transmembrane proteins and are required for sustained propagation of a synthetic cell.

Currently, the gold standard for studying cellular processes involving transmembrane proteins is detergent-based reconstitution in large unilamellar vesicles (LUVs Ø ∼100–1000 nm) (3, 5, 10). From the perspective of bottom-up synthetic cell construction, detergent-based methods have several major disadvantages. First, reconstitution takes place in LUVs, which have a low encapsulation efficiency when it comes to complex biochemical networks such as *in vitro* transcription–translation (IVTT) systems (11, 12). Second, the relatively small intraliposomal volume is too minimal for long-term sustained processes. Third, the membrane protein density and number of different membrane proteins that can be effectively co-reconstituted is limited.

The first two disadvantages can be overcome by reconstituting the membrane protein(s) of interest into giant unilamellar vesicles (GUVs, ∼Ø 1–100 μm). This can be accomplished via either a direct or an indirect route. Via the direct route, the membrane protein of interest is reconstituted into existing detergent-destabilized GUVs (13). Via the indirect route, proteins are first reconstituted in small unilamellar vesicles (SUVs Ø ∼10–100 nm) or LUVs, which are subsequently fused with empty GUVs (14), or fused into GUVs via emulsion-assisted methods (15, 16) or electroformation (17, 18). The indirect emulsion-assisted route for membrane protein reconstitution in GUVs is well-established but is still limited by the membrane protein density that can be achieved. Moreover, a synthetic cell capable of continuous growth and division has the additional requirement of continuous membrane protein insertion, which is not possible as long as insertion is ultimately reliant on detergent-destabilization.

**Figure 1.**
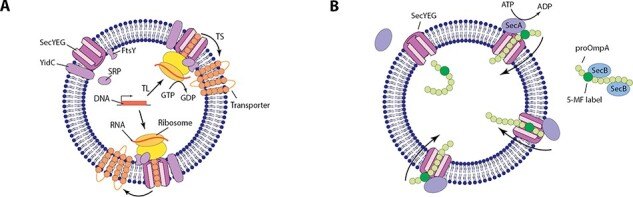
Sec-mediated insertion and translocation. (**A**) Minimal inside-out insertion system for membrane protein insertion in a synthetic cell. This involves transcription (TL) of messenger RNA from a transporter gene of interest; transertion (TS), where the ribosome docks with SecYEG and the transporter is inserted through co-translational insertion driven by GTP hydrolysis; insertases such as SecYEG and YidC; and the SRP, which targets the ribosome to the SRP receptor, FtsY. (**B**) Minimal outside-in translocation system in a synthetic cell. This involves: SecYEG as a minimal translocase; SecA, an ATPase that drives translocation; SecB, a chaperone that keeps the translocated protein in an unfolded state; a translocated protein, such as proOmpA, which for assay purposes is often labeled at position 290 with a fluorophore.

A minimal system overcoming these limitations would be a synthetic liposome incorporating a membrane protein complex that is capable of membrane protein insertion. In bacteria, this is achieved through an advanced membrane protein insertion system, the Sec translocon (19–21). While the entire system involves over 10 different (membrane) subunits, as well as the bacterial transcription and translation machinery, a minimal version would involve SecYEG and an IVTT system (Figure 1A). After transcription of a membrane protein gene of interest into messenger RNA, and translation initiation, the signal recognition particle (SRP) guides the ribosome to the SRP receptor (FtsY). As translation progresses, the peptide chain is threaded into SecYEG, which can open laterally to allow the membrane protein to insert into the lipid bilayer. Depending on the membrane protein, this process can require additional components, such as YidC.

Importantly, although non-assisted spontaneous membrane protein insertion has been observed before for a variety of proteins, such as the F-type adenosine triphosphate (ATP) synthase and membrane enzymes of the phospholipid synthesis pathway, these systems often present far lower activity compared to assisted insertion systems making them not usable for the sustained function and growth of a synthetic cell (22–24).

We therefore hypothesize that incorporation of the Sec-translocon in the membrane will act as a catalyst for the efficient insertion of newly synthesized membrane proteins, including the subunits of the Sec-translocon itself. While the aforementioned minimal system could function as the starting point for creating GUV synthetic cell compartments capable of co-translational insertion, it is still a complicated system. We hypothesized that the functional reconstitution of a minimal SecYEG-dependent translocation system could function as the starting point for creating more complex and life-like GUV synthetic cell compartments.

Here, we show a method for the reconstitution of translocationally active SecYEG into GUVs utilizing an emulsion-assisted GUV formation method developed by Göpfrich *et al*. (15). This emulsion-assisted method has previously been shown to be compatible with reconstitution of the relatively simple integrin αIIbβ3 into GUVs by fusion with empty liposomes. Here, we demonstrate that this method is also compatible with the functional reconstitution of a membrane protein complex without the addition of empty liposomes, thereby maintaining a high protein:lipid ratio. Our minimal SecGUV system shows translocation activity as tested by including the translocation-driving ATPase SecA and secretory protein substrate proOmpA (a system that has previously been shown to be active in LUVs) (Figure 1B) (25). The reconstitution of active SecYEG into GUVs establishes a promising starting point for the reconstitution of the full membrane protein insertion system for synthetic cells.

## 2. Materials and methods

### 2.1 Plasmids and strains

### 2.2 Protein purification and labeling

All strains and plasmids are listed in Table 1. SecA was overexpressed in BL21 (DE3) and purified as described previously (32). The secretory protein proOmpA was overexpressed in the temperature-sensitive *Escherichia coli* MM52, which displays translocation defects by inhibiting SecA production at 37°C. Purification and labeling with fluorescein-5-meleimide was described previously (25). Both SecYEG variants were overexpressed in BL21 (DE3). Isolated SecY-his-EG-containing membrane fractions were solubilized in 2% n-Dodecyl-$\beta$-Maltoside (DDM) and incubated with Ni^+^-nitrilotriacetic acid agarose beads (Qiagen) for 1 h. Samples were washed with 50 mM Tris-HCl pH 8, 100 mM KCl, 0.05% DDM and 40 mM imidazole and eluted by increasing the imidazole concentration to 300 mM. Labeling of single-Cys-SecYEG to Alexa Fluor 488 C_5_ Maleimide (Invitrogen) was performed during purification. Samples were solubilized as mentioned previously and washed with 100 mM KPi pH 7.4, 100 mM KCl, 0.1% DDM and 10 mM imidazole. Conjugation was performed using wash buffer containing 200 µM of fluorophore and incubated for 2 h. KPi was removed by washing with wash buffer containing Tris-HCl pH 7.4, 100 mM KCl and 0.05% DDM, and single-Cys-SecYEG was eluted with 300 mM imidazole.

**Table 1.** Strains and plasmids used in this study

Strains/plasmid	Short description	Ref.
*Strains*		
BL21 (DE3)	*F- ompT hsdSB (rB-,mB-) gal dcm (DE3)*	(26)
MM52	*F- (araD139)_B/r_ secA51(ts) Δ(argF-lac)169 λ^-^ flb-5301 Δ(fruK-yeiR)725(fruA25) relA1 rpsL150(strR) rbsR22 Δ(fimB-fimE)632(::IS1) deoC1*	(27)
*Plasmids*		
pET610	SecY-his-EG	(28)
pEK20-C313	SecY(G313C)-his-EG	(29)
pTRC99 SecA	SecA	(30)
pET502	proOmpA(C302S, C290)	(31)

### 2.3 SecYEG reconstitution

Protein reconstitution was performed in liposomes consisting of DOPC:DOPG:DOPE at molar ratio 40:30:30 with added 0.1 or 0.3 mol% DOPE-ATTO655. Chloroform stocks were mixed to the appropriate ratio, dried under N_2_ gas flow and resolubilized in Buffer A (20 mM Tris-HCl pH 7.4, 30 mM KCl, 2 mM DTT). To obtain SUVs, multilamellar vesicles (MLVs) were diluted to 5 mM total lipid concentration and sonicated on ice (5 × 5 s, 4 µm amplitude). Lipids were solubilized in 0.5% Triton X-100 and mixed with purified SecYEG to obtain a 1:500 SecYEG:lipid ratio. The eventual SecYEG:lipid concentration in SecGUVs is assumed to be the same ratio. Triton was removed by adding and refreshing bio-beads in several rounds (2 × 2 h, 1 × 16 h overnight, final 1 × 1 h). Proteoliposomes were obtained by ultracentrifugation (TLA 110, 550.000 RCF, 30 min, 4°C) and resolubilized in Buffer A to an estimated concentration of ∼10 mM lipids. The concentration of recovered liposomes was determined by liquid chromatography-mass spectrometry (LC–MS).

### 2.4 GUV formation

GUVs were formed based on the work by Göpfrich *et al*. (15). Contrary to their protocol, our proteoSUVs (membrane protein-containing SUVs) were not diluted with regular SUVs. While adding regular SUVs might increase the overall GUV yield, it brings down the SecYEG concentration in the membrane, which is undesirable given the low translocation turnover of SecYEG. Care should be taken to keep all buffers that contain SUVs or GUVs equiosmolar to the later used reaction conditions. Briefly, proteoSUVs or regular SUVs were diluted to a concentration of 3 mM in suitable buffer (typically 20 mM Tris pH 7.4, 2 mM DTT, 10–100 mM KCl) and extruded seven times through a 100-nm pore size polycarbonate filter (Avestin). It is important to use a 100-nm pore size filter rather than a 50-nm pore size filter, both for ease of extrusion and because some phospholipid loss can occur when extruding with a 50-nm pore size filter (Supplementary Figure S12). After extrusion, SUVs were diluted to a final concentration of 2.5 mM in the same buffer, with 100 mM sucrose and 10 mM MgCl_2_ added. A fresh oil solution was prepared consisting of 1.4 wt% 008FS (RAN Biotechnologies), 10.5 mM Krytox (157FS(H), DuPont), in HFE7500 (3 m), using dried and degassed HFE7500. For a typical sample, 100 µl of the SUV solution was added to 200 µl oil solution and vortexed vigorously for 20 s. The emulsion was incubated overnight at 4°C. For release, 100 µl of an equiosmolar release buffer (20 mM Tris-HCl pH 7.4, 2 mM DTT, 100 mM glucose, 40–130 mM KCl) was added, as well as 50 µl of a 30 *v/v*% perfluoro-1-octanol (PFO) solution in HFE7500. Immediately following PFO addition, the sample was shaken four times and incubated for 5 min at room temperature. An aqueous layer which contains the GUVs forms on top of the sample, which is carefully harvested using a pipette. In order to further concentrate the GUVs, the collected sample was centrifuged (3000 RCF, 10 min), the supernatant is discarded and the (invisible) pellet is resuspended in a small volume of equiosmolar concentration buffer (typically 20 mM Tris-HCl pH 7.4, 2 mM DTT, 100–200 mM KCl).

The GUVs were tested for unilamellarity using alpha hemolysin (25 µg/ml) and calcein (10 µM). Briefly, αH (Sigma-Aldrich) was solubilized in milliQ. Subsequently, αH and calcein, or calcein only, were added to the GUV sample and the sample was incubated for 1 h at room temperature. The ratio of the inner (I_in_) and outer fluorescence intensity (I_out_) was determined using ImageJ, where a unilamellar membrane allows for αH pore formation and calcein transport, leading to an I_in_/I_out_ close to 1.

### 2.5 Confocal microscopy

Confocal microscopy was employed to estimate GUV yield and quality at various stages of the formation protocol. Typically, a 10-µl sample was taken and observed on a glass slide (6 Channel μ-Slide glass bottom, Ibidi). The glass surface was passivized by incubating the slides with a 5 *w/v*% polyvinyl alcohol (PVA) solution for a minimum of 3 h at room temperature. After incubation, the slides were cleaned with milliQ and dried with nitrogen gas. GUV formation and SecGUV quality control were performed at room temperature on a Leica SP8 Liachroic confocal microscope (655 nm fixed line emission, 665–800 nm emission using a Hybrid detector in counting mode) or a Zeiss LSM 710 confocal microscope (655 nm fixed line emission, 665–800 nm emission using a photomultiplier tube) using a 40× objective or a 63× oil objective. Images were analyzed using ImageJ.

### 2.6 LC–MS analysis

Lipid concentrations were determined using an Accela1250 HPLC system coupled with an ESI–MS Orbitrap Exactive (ThermoFisher Scientific) as described by de Kok *et al*. (33). In short, samples were prepared by applying 150 µl distilled 1-butanol, mixing by vortex and separating by centrifugation (16.000 RCF, 2 min), and this process was repeated once more. The lipid layer containing 1-butanol was collected in glass vials and dried under a nitrogen gas stream. Lipid films were subsequently dissolved in methanol and injected on a Waters ACQUITY Premier CSH C18 (1.7 µm, 2.1 × 150 mm) column. Lipid separation was achieved by applying a changing gradient of eluent A: MQ:MeCN (40:60) containing 5 mM ammonium formate and eluent B: MQ:MeCN:1-BuOH (0.5:10:90) also containing 5 mM ammonium formate. Mass spectrometry specifications and settings have been described previously (34). Thermo Scientific XCalibur processing software was used to analyze spectral data by applying the Genesis algorithm-based automated peak area detection and integration. Total ion counts for each extracted lipid were normalized for the internal standard (10:0 PG).

### 2.7 Translocation gel assay

Successful SecYEG-GUV formation was assessed by performing a fluorescently labeled proOmpA translocation assay as described previously (25). In short, SUVs/GUVs were incubated with 0.15 µM labeled-proOmpA at 37°C for 30 min in a buffer containing 20 mM Tris-HCl pH 7.4, 30 mM KCl, 10 mM DTT, 0.1 mg/ml bovine serum albumin (BSA), 50 µg/ml creatine kinase, 10 mM phosphocreatine, 0.25 µM SecA, 0.5 µM SecB and 2 mM ATP-Mg^2+^. Samples were treated with proteinase K (0.1 mg/ml) for 15 min on ice and translocated proOmpA was subsequently precipitated by the addition of up to 8% (*w/v*) trichloroacetic acid (TCA) and pelleted by centrifugation (17.000 RCF, 15 min) and washed twice with acetone, resuspended in sodium dodecyl sulphate buffer and analyzed using sodium dodecyl sulphate–polyacrylamide gel electrophoresis (SDS–PAGE).

### 2.8 Translocation confocal assay

The confocal version of the proOmpA translocation assay works according to the same principle as the translocation gel assay. Briefly, after SecGUV release, a single sample of 100 µl SecGUV is concentrated in 10 µl of concentration buffer as described above (GUV formation). The concentrated SecGUVs are kept on ice. A translocation reaction mix is prepared, such that upon the addition of 10 µl of concentrated SecGUVs, the final reaction component concentrations are: 0.15 µM labeled-proOmpA, 20 mM Tris-HCl pH 7.4, 30 mM KCl, 10 mM DTT, 0.1 mg/ml BSA, 50 µg/ml creatine kinase, 10 mM phosphocreatine, 0.25 µM SecA, 0.5 µM SecB and 2 mM ATP-Mg^2+^. The 2 mM ATP-Mg^2+^ is added last, once the entire reaction mixture has been prepared, in order to start the reaction. The negative control lacks SecA. The reaction mixture is loaded into a reaction chamber consisting of a microscopy grade rectangular coverslip (26 × 76 mm, #1.5, Epredia), a two-sided spacer sticker (SecureSeal, 25 × 25 mm, 0.12 mm depth, Grace Biolabs) and a round glass coverslip to close the chamber (26 mm, Avantor). The rectangular coverslip and spacer are assembled beforehand and the area inside the spacer is incubated with 5 wt% PVA for passivation as described above (Confocal microscopy). Once the sample is loaded and the chamber is closed, the slide is placed in a temperature-controlled Leica SP8x confocal microscope set at 37°C. Temperature control consisted of heated air circulated in a Perspex box around the main body of the microscope. To follow the GUVs, DOPE-ATT655 was excited at 638 nm using a continuous white-light laser, with emission collected at 650–800 nm using a Hybrid detector in counting mode. To follow translocation, labeled-proOmpA was excited at 488 nm using a continuous white-light laser, with emission collected at 494–600 nm using a Hybrid detector in counting mode. Images were taken every 10 min. Image analysis was performed using ImageJ. Importantly, only SecGUVs that were in focus, non-deflated and had no major deformations were taken along in the analysis (Supplementary Figure S13 for details). A script was used to identify and number viable GUVs, as well as measure their diameter (Supporting information S14). Subsequently, the viability of the identified SecGUVs and the intensity measurements were assessed manually.

## 3. Results and discussion

**Figure 2.**
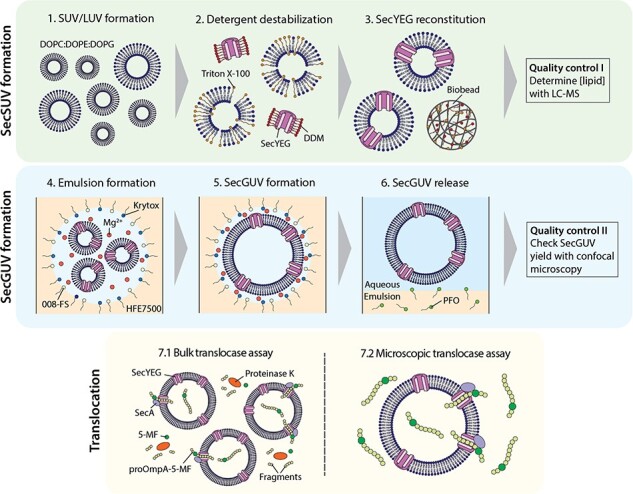
General approach and method. The method for SecGUV formation has seven major steps. First, an SUV/LUV sample with the desired lipid composition is prepared. Second, SecYEG is isolated using DDM and mixed with Triton X-100 destabilized SUVs/LUVs. Third, as the detergents are slowly extracted using biobeads, the SecYEG is inserted into the SUV/LUV membrane, creating SecSUV/LUVs. Fourth, a water-in-oil emulsion is prepared using extruded SecSUVs (Ø 100 nm). The hydrophilic anionic headgroups of Krytox create a negatively charged water–oil interface. This attracts Mg^2+^ to the interface, which in turns attracts SecSUVs to the interface due to 30 mol% of the anionic phospholipid DOPG present in the SUV membrane. Fifth, the SecSUVs fuse into SecGUVs at the water–oil interface. Sixth, SecGUVs are released through addition of the emulsion destabilizing agent PFO. Seventh, translocase activity of the SecGUVs is first tested in the more sensitive bulk version of the assay. Here, 5-MF proOmpA is translocated across the GUV membrane by SecYEG. Any proOmpA-5-MF that is left on the outside of the SecGUVs is broken down by proteinase K. Only the protected proOmpA-5-MF is isolated through precipitation and visualized using SDS–PAGE and fluorescent imaging. Alternatively, translocation is observed in real time using confocal microscopy.

The functional reconstitution of the minimal translocation system described above involves a number of steps (Figure 2). First, SecSUVs/LUVs are formed by a well-established reconstitution method. Briefly, MLVs are made unilamellar by sonication (Step 1), destabilized by the detergent Triton X-100 (Step 2), supplemented with purified SecYEG in detergent and re-stabilized by the removal of detergent by successive rounds of Bio-Bead incubation (Step 3). The formed SecSUVs/LUVs are extruded to the correct SecSUV size (∼Ø 100 nm) and subsequently added to the aqueous phase of a Krytox-stabilized water-in-oil emulsion (Step 4). At the water–oil interface, the negatively charged carboxylic acid headgroup of the Krytox surfactant attracts Mg^2+^ (14, 35, 36). The Mg^2+^ allows SecSUVs containing anionic phospholipids to fuse into SecGUVs at the water–oil interface (Step 5). SecGUVs are released through the addition of an emulsion-destabilizing agent, PFO (Step 6). SecYEG activity can be assayed by observing the translocation of a fluorescently labeled secretory protein, in this case proOmpA, employing either SDS–PAGE for a bulk measurement (Step 7.1) or by confocal microscopy for individual observation (Step 7.2).

### 3.1 SecYEG reconstitution in GUVs

Our first objective was to investigate whether we could produce unilamellar GUVs using the optimal lipid composition for SecYEG activity. As with many bacterial inner membrane proteins (37), the lipid environment is essential for optimal functioning of the Sec translocon with a requirement for both acidic phospholipids (38) and non-bilayer lipids (39). We therefore opted to form the SecGUVs using lipid compositions previously established to be highly functional namely: 1,2-dioleoyl-sn-glycero-3-phosphatidylcholine (DOPC), 1,2-dioleoyl-sn-glycero-3-phosphatidylglycerol (DOPG) and 1,2-dioleoyl-sn-glycero-phosphatidylethanolamine (DOPE) in a molar ratio of 40:30:30 (40). As the conical shape of unsaturated phosphatidylethanolamine can destabilize bilayer formation when used at higher concentrations, inclusion of DOPE could possibly interfere with GUV formation using the emulsion-assisted method (41, 42). However, initial experiments showed that the emulsion-assisted method is compatible with a high PE ratio (Supplementary Figure S1). In order to test whether these GUVs were unilamellar and non-leaky, we added the pore-forming protein alpha-hemolysin and the small fluorescent dye calcein (15, 43). In the presence of alpha-hemolysin, calcein can enter the GUVs, whereas in the absence of alpha-hemolysin, the GUVs remain almost completely dark (Supplementary Figure S2A). Some increase in signal can be observed in the negative control, suggesting that not all GUVs that are formed are free of defects, allowing some calcein to leak in over time (Supplementary Figure S2B). Together, these results show that the emulsion-assisted method is compatible with the formation of GUVs containing high concentrations of DOPE.

Next, we attempted the formation of SecGUVs from SUVs containing SecYEG (Figure 2, Steps 2–6). As reported previously, there are several factors that influence (proteo)GUV yield. These include the SUV concentration, the freshness of the various solutions used to prepare the emulsion and the release method (15). Indeed, by determining the precise lipid concentration of the SecSUVs using LC–MS (33), we could confirm that the starting lipid concentration has a major impact on SecGUV yield, with an optimal starting concentration of ∼2.5 mM (Supplementary Figure S3). SecGUV release, which can be done in one of two ways, had a similar effect. Either the emulsion-destabilizing agent PFO and release buffer are added and allowed to slowly dissolve the emulsion, or the entire mixture is strongly shaken for several seconds immediately following addition of the destabilizing agent and the release buffer. A higher SecGUV yield was observed when using the shaking release method (Supplementary Figure S4). In general, SecGUV yield was lower than regular GUV yield and showed greater variability. This difference can be explained by the presence of the SecYEG complex in the lipid bilayer, which likely makes the SecSUVs less fusogenic. The variability in SecGUV yield itself can be explained by differences in the quality of the SecSUVs used as starting material. However, even when using the exact same solutions for SecGUV formation, this could result in substantial yield variability that we could not readily explain (results not shown). We found that the temperature at the moment of release also influences GUV yield, with a lower yield at 4°C as compared to room temperature (Supplementary Figure S5). This underscores the importance of allowing the emulsion to settle at room temperature before attempting release. One noteworthy difference between our approach and that of Göpfrich *et al*. is that they supplemented their proteoSUVs (membrane protein-containing SUVs) with a 10-fold addition of regular SUVs before proteoGUV formation (15). In order to obtain higher protein:lipid ratios that are often highly beneficial for the overall activity during assays, regular SUVs were not included in our protocol.

**Figure 3.**
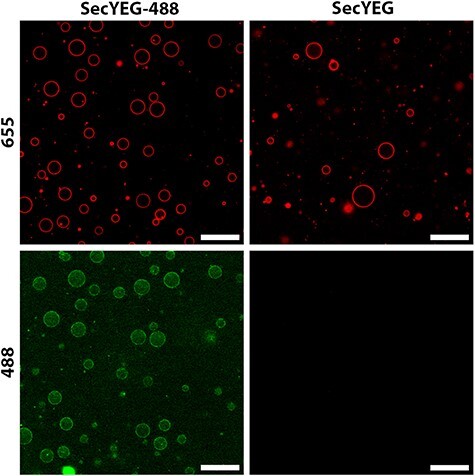
Formation of SecGUVs from SecSUVs containing SecYEG-AF488. Differences in GUV quantity are due to random variation within the field of view. Lipid composition DOPC:DOPE:DOPG 40:30:30 supplemented with 0.1 mol% DOPE-ATTO655. Red 655 channel: DOPE-ATTO655. Green 488 channel: SecYEG-488. Scale bars: 50 µm.

Taking the above considerations into account, we were able to show the formation of SecGUVs from SecSUVs (Figure 3). To confirm the localization of SecYEG in the GUV membrane, we used modified SecY-148C-EG tagged with Alexa Fluor 488 C_5_ maleimide. SecY-148C-EG was present within nearly every formed GUV, although some variation in intensity between SecGUVs could be observed.

**Figure 4.**
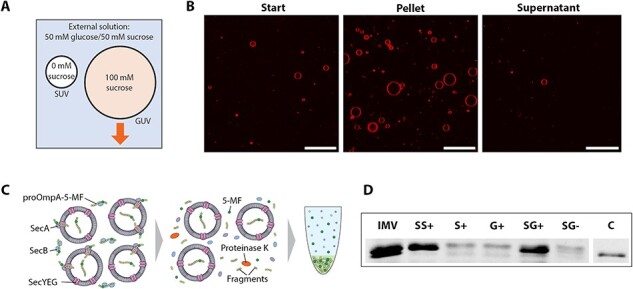
SecGUV concentration and translocation assay. (**A**) Density-based concentration of GUVs, but not SUVs (not to scale). (**B**) Start: typical SecGUVs starting concentration after diluting the sample in concentration buffer (see ‘Materials and methods’ section). Pellet: SecGUVs can be pelleted and dissolved in a small volume to increase the concentration. Supernatant: pelleting leaves almost no SecGUVs in the supernatant. Scale bar: 50 µm. (**C**) Schematic representation of the bulk assay. After translocation, remaining proteins on the outside of the SecGUVs are broken down by proteinase K. Upon TCA precipitation, protected proOmpA-5-MF precipitates, but free 5-MF dye does not. The precipitated proOmpA-5-MF can be redissolved and put on an SDS–PAGE gel for visualization. (**D**) SDS–PAGE gel showing proOmpA-5-MF translocation in SecGUVs. All reactions took place in presence of the full reaction mix with or without SecA. IMV = inner membrane vesicles; SS+ = SecSUVs in complete reaction mixture; S+ = regular SUVs in complete reaction mixture; G+ = regular GUVs in complete reaction mixture; SG+ = SecGUVs in complete reaction mixture; SG− = SecGUVs in reaction mixture without SecA; C = proOmpA-5-MF control at the end of the gel represents 5% of the proOmpA-5-MF added to the reactions to check if it was still fluorescent.

To provide enough material for functional assays and to improve assay quality, we implemented an additional density-based concentration step, thereby concentrating the SecGUVs. By adding 100 mM sucrose to the inside of the SecGUVs during emulsion preparation and by keeping the outside solution equiosmolar using lower molecular weight solutes, SecGUVs could be pelleted through centrifugation under mild conditions (see ‘Materials and methods’ section). When the SecGUV pellet was dissolved in a small volume (typically ∼10 μl), a more concentrated SecGUV sample was obtained (Figure 4A and B). This concentration effect was confirmed using LC–MS, which showed that for both regular GUVs and SecGUVs, most of the starting phospholipid material ended up in the pellet and only a small amount in the supernatant (data not shown). Using these data, we also estimated the SecGUV release efficiency in terms of the phospholipid concentration. Using the LC–MS approach described in the ‘Materials and methods’ section, we found a maximum phospholipid concentration of 0.5 mM, although typically less material was released. As the starting SecSUV concentration was 2.5 mM phospholipids, this implies a maximum release efficiency of 20%.

### 3.2 SecGUV bulk activity assay

To examine the activity of SecGUVs, we applied a fluorescence-based translocation assay reported previously (Figure 2, Step 7.1) (25). Compared to other membrane transporters, the *in vitro* transport rate of the Sec translocon is low in the absence of a proton motive force. In case of the translocation of the secretory protein proOmpA, a turnover of 0.1 s^–1^ has been reported (44). This is consistent with more recent measurements, which put the average processivity of SecYEG-mediated translocation in proteoliposomes at 40 amino acids per second (45). Hence, the activity of SecGUV is more readily observed in bulk as compared to translocation into individual SecGUVs.

Briefly, in this bulk assay, fluorescein-5-maleimide (5-MF)-labeled proOmpA is translocated into the SecGUVs in the presence of SecA and ATP, whereupon the presence of residual fluorescent proOmpA is determined after proteinase K digestion as analyzed on SDS–PAGE (Figure 4C). Only successfully translocated proOmpA-5-MF will be inaccessible to the externally added proteinase K. For the assay, we mixed the concentrated SecGUVs and various positive and negative controls with the reaction mixture and examined the translocation of proOmpA-5-MF (Figure 4D). The first two lanes represent the two positive controls, which are *E. coli* inner membrane vesicles (IMVs) and SecSUVs mixed with the full reaction mixture, which includes SecA and ATP. Both the IMVs and SecSUVs show the expected translocation of proOmpA. The IMV sample shows a second band due to the processing of proOmpA into OmpA by the leader peptidase which is also present in the IMVs. The next two lanes represent the two negative controls, which are empty SUVs and GUVs, respectively, both mixed with the full reaction mixture. Only a weak fluorescent background is observed. The next two lanes represent the SecGUVs mixed into the reaction mixture with and without SecA. The final band represents a pure proOmpA-5-MF control. Indeed, only the SecYEG- and SecA-containing samples showed proOmpA-5-MF protected from proteinase K digestion after translocation. As was observed in the density-based concentration step, only GUVs and SecGUVs, but not SUVs or SecSUVs, were collected and used. This implies that the activity we observed is due to SecGUVs and does not come from contaminating SecSUVs.

### 3.3 Active proOmpA translocation in SecGUVs

Fluorescence microscopy revealed that the SecGUVs inside the reaction mixture showed substantial aggregation after 30 min incubation in the reaction mixture (Supplementary Figure S6). The aggregate formation could reduce translocation efficiency as well as interfere with direct observations during microscopy analysis. We ascertained that the aggregation is primarily caused by the ATPase SecA, which is known to facilitate membrane–membrane interactions *in vitro* (Supplementary Figure S7A) (46, 47). This aggregation was prevented by the addition of 50 mM L-arginine (Supplementary Figure S7B), which by itself does not have effect on the proOmpA translocation efficiency (Supplementary Figure S8).

**Figure 5.**
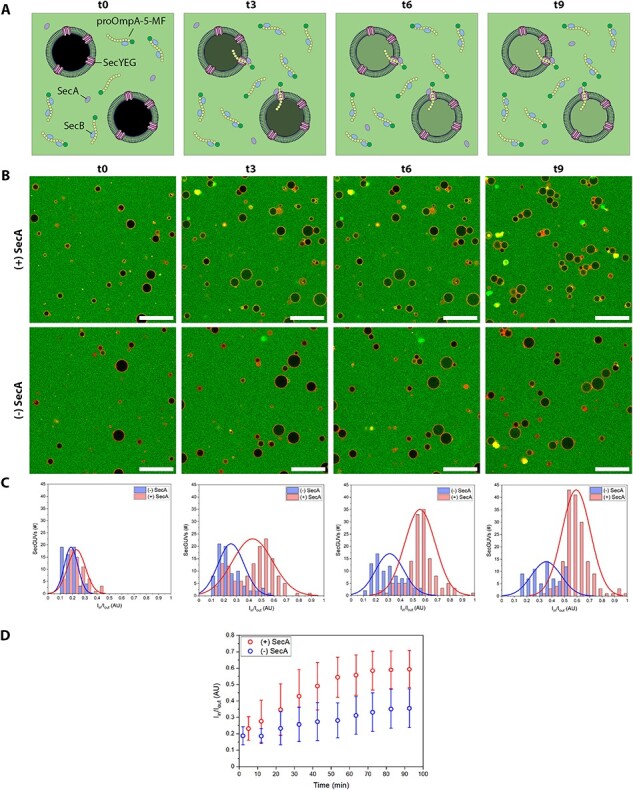
Active translocation of proOmpA into SecGUVs. (**A**) Schematic representation of the uptake of proOmpA-5-MF into SecGUVs in the presence of SecA over time. (**B**) Overlay fluorescence images of time points ∼0, 30, 60 and 90 min after the reaction has been started. SecGUVs in the total reaction mix with or without SecA. Red channel: DOPE-ATTO655. Green channel: proOmpA-5-MF. Scale bars: 50 µm. (**C**) Histograms of fluorescence intensity ratio I(in)/I(out) distributions. Reaction conditions with and without SecA. (**D**) Plot of fluorescence intensity ratio change over time. Error bars indicate sample standard distribution. For the averages of each time point, *P*-value <0.001 based on an unequal variances *t*-test.

Next, we moved on to the direct observation of proOmpA-5-MF translocation across the SecGUV membrane by making use of confocal microscopy (Figure 2, Step 7.2). Active translocation can be measured in terms of the ratio between the fluorescence intensity inside and outside the SecGUVs (I_in_/I_out_) (Figure 5A). As more proOmpA-5-MF is transported inside of the SecGUVs, this ratio increases. We prepared multiple batches of SecGUVs, which were concentrated, combined and split over two reaction mixtures, one with and one without SecA. Even at a proOmpA-5-MF concentration as low as 150 nM and despite the low translocation rate of SecYEG, we were able to measure an increase in fluorescence to the inside of the SecGUVs over time (for measurement details, see ‘Materials and methods’ section). As can be observed, the SecGUV populations with and without SecA show a clear difference in fluorescence intensity inside the SecGUV lumen (Figure 5B). Moreover, when comparing the two populations over time, we can see that the fluorescence intensity ratio distributions separate (Figure 5C, see Supplementary Figure S9 for all time points). The general increase in total number of SecGUVs present in the images and histograms is due to more SecGUVs sinking down to the surface of the observation chamber over time (see Supplementary Table S10). Finally, when we plot the average fluorescence intensity ratio and sample error bars over time, we can see a clear increase in the fluorescence intensity ratio in the SecA-containing sample compared to the sample which lacks SecA (Figure 5D, for data overview see Supplementary Table S10). Importantly, this effect is not due to differences in the size distribution of the SecGUVs that have been analyzed, as these distributions are mostly similar (Supplementary Table S10, Supplementary Figure S11). Notably, we also observed an increase in the fluorescence intensity ratio of the sample without SecA that is limited to some GUVs. This can be explained by the slight leakiness of the GUVs formed with the emulsion-assisted method (Supplementary Figure S2B), which is likely to be higher when pure proteoliposomes are used as starting material because of the presence of membrane proteins as well as some residual detergent. Finally, translocation is a slow process, which is due in part to the low turnover of SecYEG, but also due to the fact that SecGUV samples were kept on ice until the reaction was started, at which point these needed to warm up to 37°C within the temperature-controlled microscopy set-up (see ‘Translocation confocal assay’ section). However, combined with the previous results (Figures 3 and 4D), the confocal assay shows that SecYEG is responsible for the translocation of proOmpA-5-MF into the SecGUVs.

## 4. Conclusions

Here, we have shown that the core complex of the Sec translocon, SecYEG, can be functionally reconstituted into GUVs using the emulsion-assisted GUV formation method. SecGUVs actively translocate the unfolded secretory protein proOmpA across the SecGUV membrane. There are many different methods for GUV formation, each with its own advantages and disadvantages (14, 16, 43, 48–56). Many of these methods are complicated or only partly compatible with membrane protein reconstitution. Contrary to these approaches, we expanded and optimized the emulsion-assisted method, and we have shown that this method is compatible with the reconstitution of a multisubunit integral membrane protein, which is a major step forward in the functionalization of synthetic cell membranes.

Currently, our system is capable of translocation. The next step would be to show that we can insert a functional membrane protein into the GUV membrane via a minimal Sec-dependent route (Figure 1A). This would require the presence of an IVTT system, as membrane protein insertion relies on translocation coupled to insertion (21). Successful insertion and folding of many interesting membrane proteins, such as those involved in membrane biogenesis (5) or energy metabolism (3), would almost certainly also require other components of the insertion machinery to be present such as SRP (Ffh) and the SRP receptor (FtsY) (57). One particularly important protein is the membrane insertase YidC, which can form a transient complex with SecYEG, thereby aiding the insertion process (58).

Finally, an important feature of membrane protein insertion in living cells is that it proceeds from inside the cell. Previous work has shown that the emulsion-assisted GUV formation method can be used to encapsulate complex mixtures (15, 16). Either a lysate-based IVTT system as employed here or a cell-free system based on purified components (59) could be encapsulated using the emulsion-assisted method to mimic membrane protein insertion from inside the synthetic cell. Should IVTT encapsulation prove incompatible with the one-step emulsion-assisted method, an alternative approach would be to opt for the two-step process described by Weis *et al*. on which the one-step approach is originally based (16). Thus, the next step is to go from translocation to insertion.

## Supplementary Material

ysae007_Supp

## Data Availability

All relevant data are provided in the main text and supplementary information.

## Material availability

Plasmids are available through the corresponding author. Other materials used in this work are widely available as indicated in the ‘Materials and methods’ section.
