# Supplementary material for: SecYEG-mediated translocation in a model synthetic cell
Source: Synth Biol (Oxf). 2024 May 10;9(1):ysae007. doi: 10.1093/synbio/ysae007 (PMC11131593; doi:10.1093/synbio/ysae007)
Supplement: ysae007_Supp [file ysae007_supp.zip › supporting_information.pdf]

Supporting Information

SecYEG-mediated Translocation in a Model Synthetic Cell

Ludo L.J. Schoenmakers†,1, Max J. den Uijl†,2, Jelle L. Postma3, Tim A.P. van den Akker2, Wilhelm T.S. Huck1, Arnold J.M. Driessen2

1 Radboud University, Institute of Molecules and Materials, 6525 AJ Nijmegen, The Netherlands
2 University of Groningen, Groningen Biomolecular Sciences and Biotechnology, 9747 AG Groningen, The Netherlands
3 Radboud University, General Instrumentation, 6525 AJ Nijmegen, The Netherlands

Corresponding authors:
Arnold J.M. Driessen – email: a.j.m.driessen@rug.nl
Wilhelm T.S. Huck – email: w.huck@science.ru.nl

Contents

1. GUV characterization, release, and yield 2
- S1: Effect of DOPE molar concentration on GUV yield and quality 2
- S2: Unilamillarity check of DOPE-containing GUVs 2
- S3: Effect of SecSUV lipid concentration on SecGUV yield 3
- S4: Regular versus shaking GUV release. 4
- S5: Comparison of GUV yield at different release temperatures 4

2. GUV and SecGUV aggregation 5
- S6: GUV and SecGUV aggregation in the translocation reaction mix. 5
- S7: Effect of arginine and spermidine on GUV and SecGUV aggregation 6
- S8: Effect of arginine and spermidine on proOmpA translocation 7

3. Confocal translocation assay 8
- S9: Overview of histograms of the intensity ratios I(in)/I(out) 8
- S10: Overview of SecGUV counts, average intensities, and sizes per sample per time point 9
- S11: Graphic representation of size distributions of SecGUVs per time point in each sample 9
- S12: Effect of number of extrusion passes on lipid loss 10
- S13: Selection criteria for SecGUV intensity ratio measurements 10

4. SecGUV detection and size determination 11
- S14: Script for SecGUV identification and size determination 11

1. GUV characterization, release, and yield

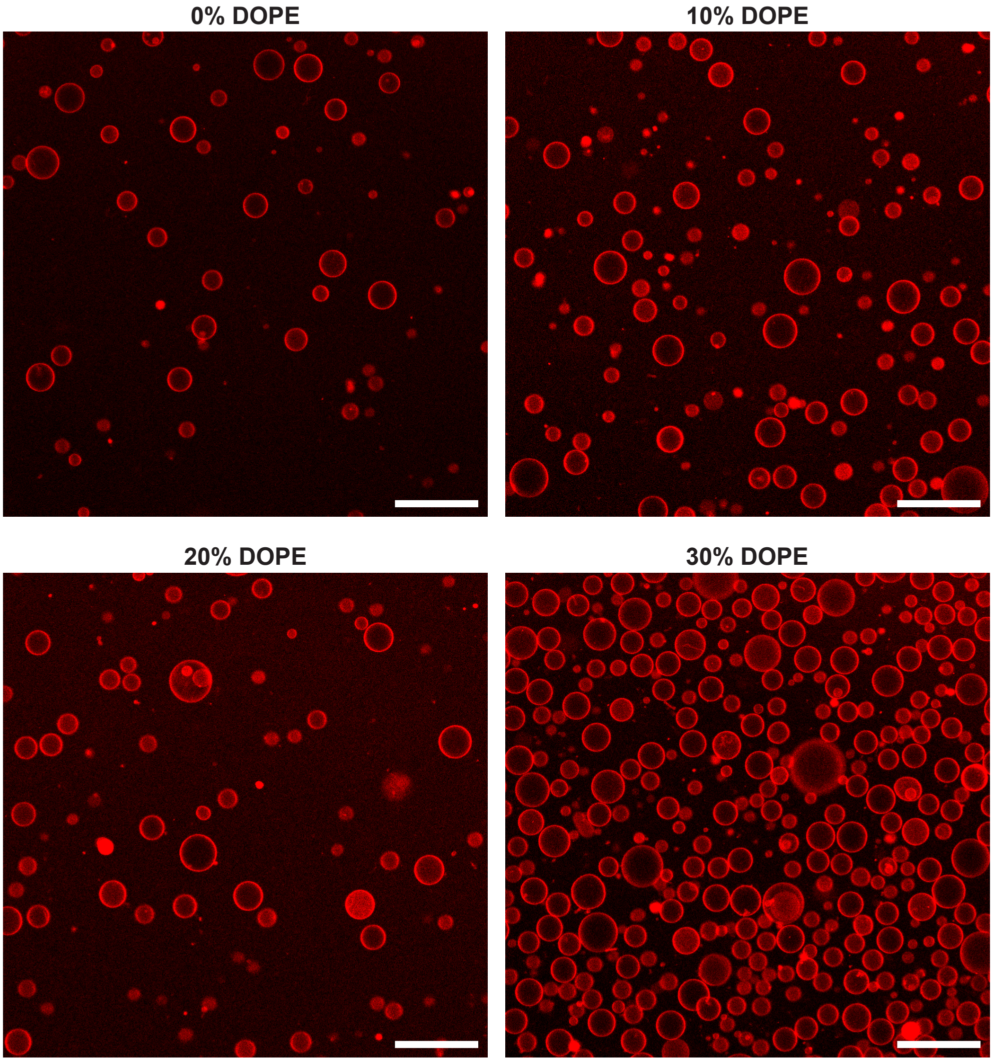

Figure S1. Effect of DOPE molar concentration on GUV yield and quality. Confocal images of GUVs with lipid composition DOPC:DOPG:DOPE with DOPG fixed at 25% and DOPC, DOPE at variable concentrations, supplemented with 0.1 mol% DOPE-ATTO655 (red channel). As can be observed, yield can vary significantly from sample to sample. Scale bars 50 µm.

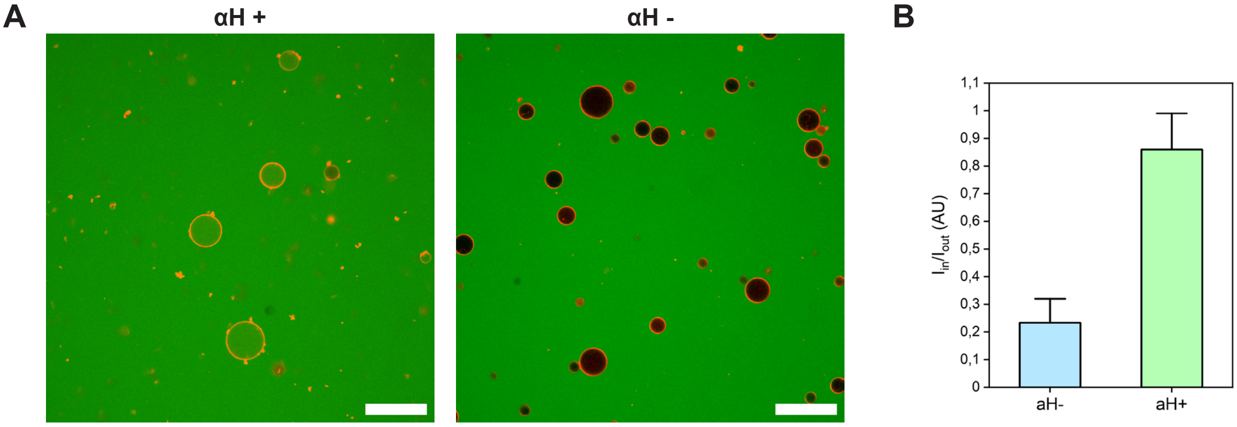

Figure S2. Unilamillarity check of DOPE-containing GUVs. (A) GUVs with lipid composition DOPC:DOPG:DOPE 40:30:30 with added 0.1 mol% DOPE-ATTO655. Overlay images of conditions with 10 µM calcein and 25 mg/ml alpha hemolysin (αH+) and with only calcein (αH-). Images taken after 1h incubation at room temperature. Red channel = DOPE-ATTO655. Green channel = calcein. Scale bars 50 µm. (B) Histogram of fluorescence intensity ratio I(in)/I(out) showing the difference between GUVs with and without added alpha hemolysin.

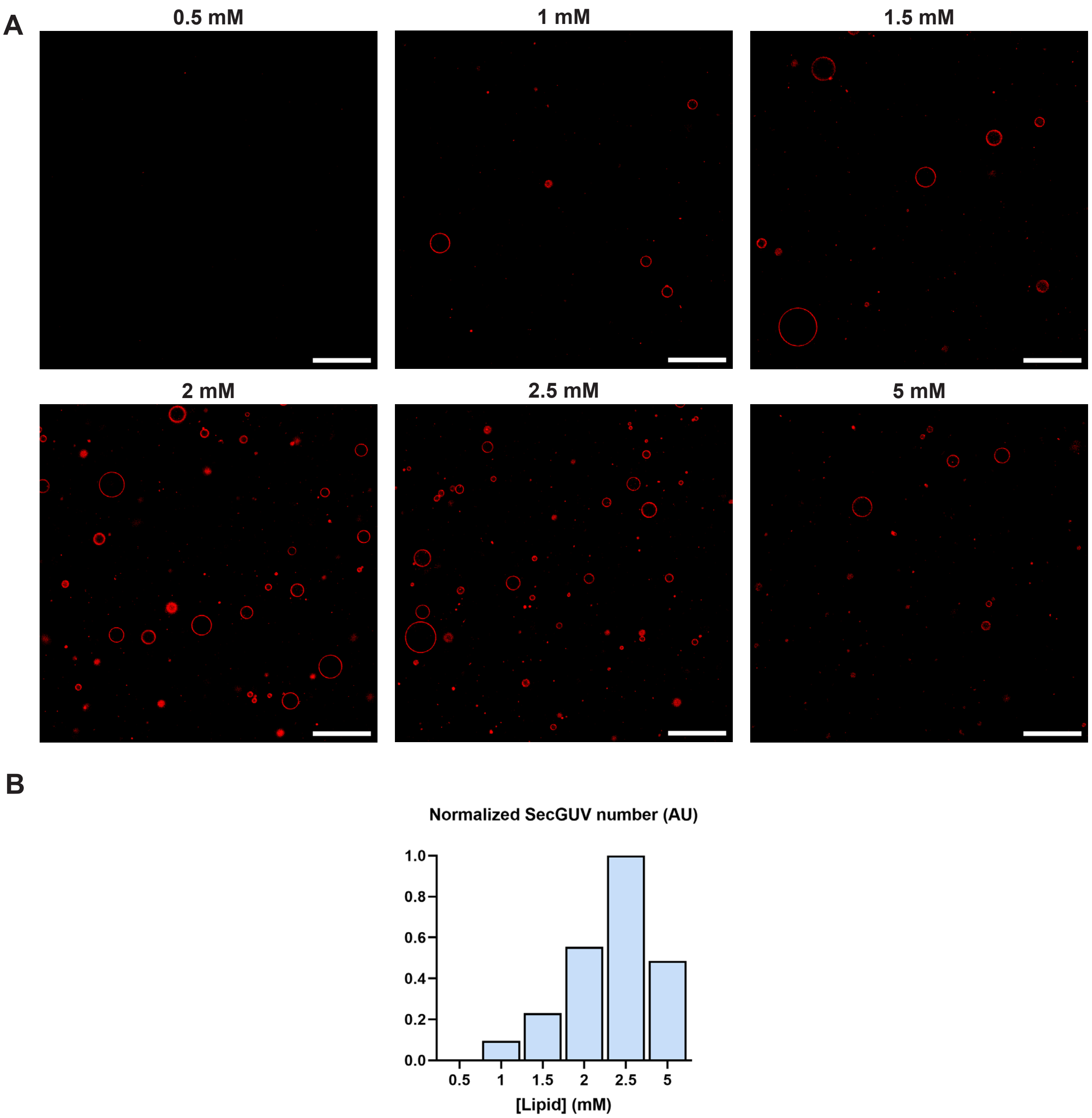

Figure S3. Effect of SecSUV lipid concentration on SecGUV yield. (A) Confocal images of SecGUVs produced with SecSUVs with lipid composition DOPC:DOPG:DOPE 40:30:30 with added 0.1 mol% DOPE-ATTO655 and a protein:lipid ratio of 1:500. Scale bars 50 µm. (B) Estimate of relative SecGUV yield for different SecSUV lipid starting concentrations. SecGUVs were counted from z-stacks of sample volumes taken from representative areas of each sample.

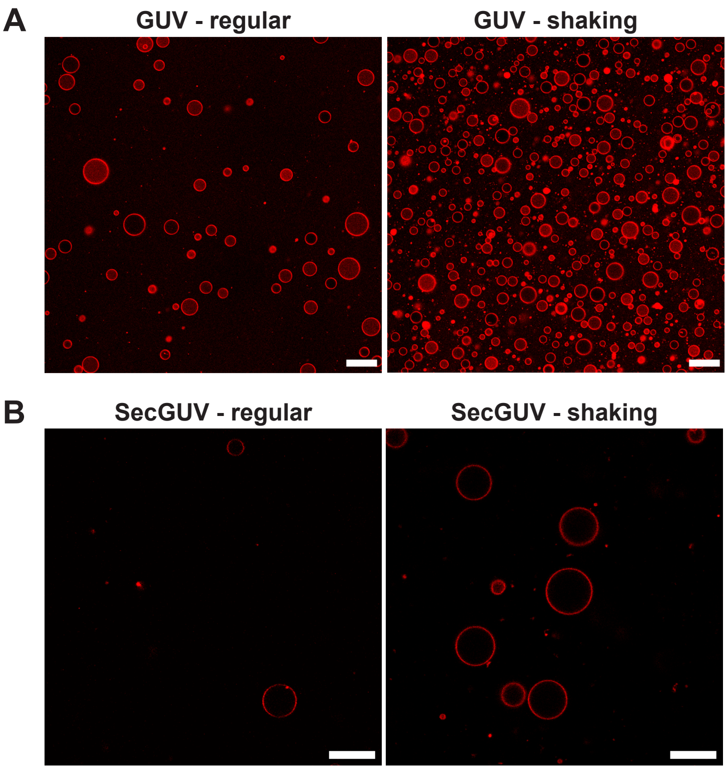

Figure S4. Regular versus shaking GUV release. (A) Typical field of view after GUV release with composition DOPC:DOPG:DOPE 40:30:30 supplemented with 0.1 mol% DOPE-ATTO655 (red channel). Scale bars 50 um. (B) Typical field of view after release of SecGUVs with composition DOPC:DOPG:DOPE 40:30:30 supplemented with 0.1 mol% DOPE-ATTO655 (red channel) and with a protein:lipid ratio of 1:500. Scale bars 20 µm. As can be observed, yield is significantly lower when starting with proteoSUVs instead of SUVs.

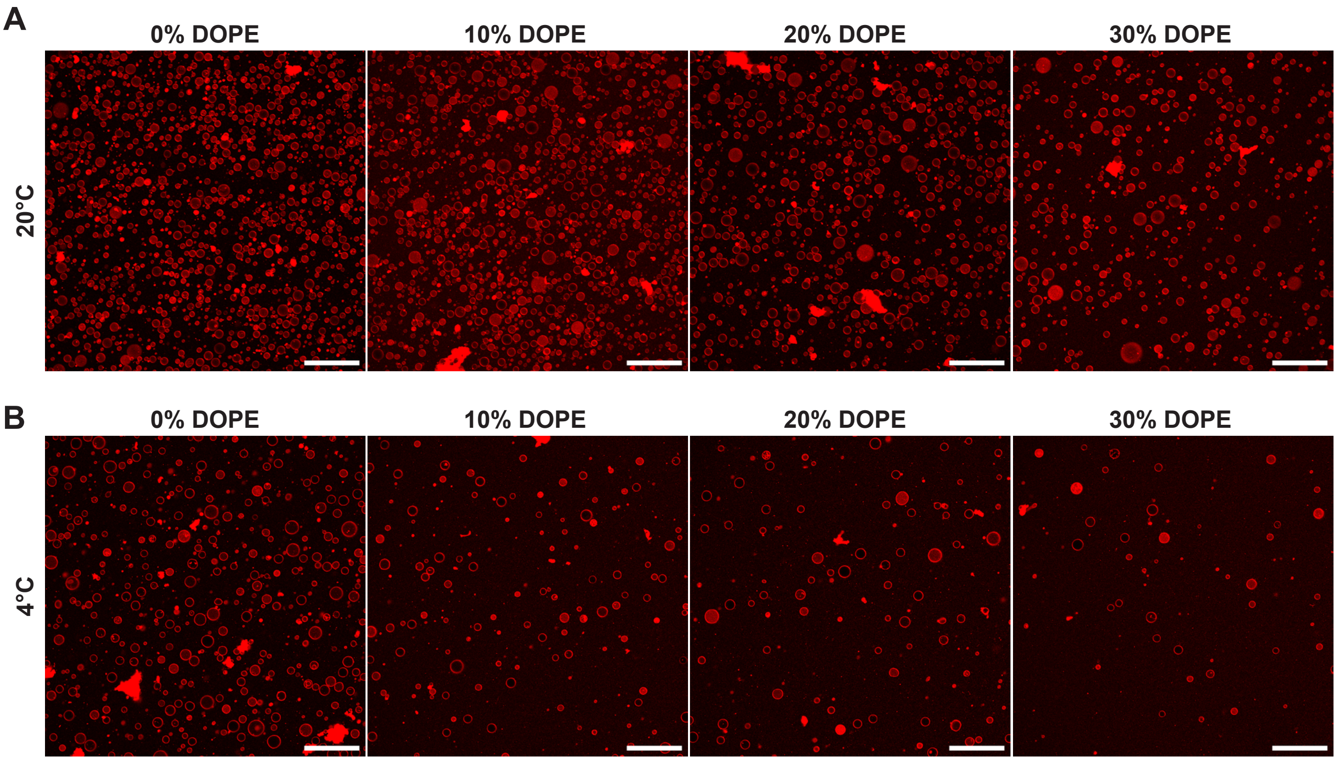

Figure S5. Comparison of GUV yield at different release temperatures. Lipid composition POPC:POPG:DOPE with POPG fixed at 25% and POPC, DOPE at variable concentrations. Supplemented with 0.1 mol% DOPE-ATTO655 (red channel). (A) Release at 20°C. As the DOPE concentration increases, yield decreases. (B) Release at 4°C, which results in a lower yield than at 20°C, again dependent on the DOPE concentration. Scale bars 100 µm.

2. GUV and SecGUV aggregation

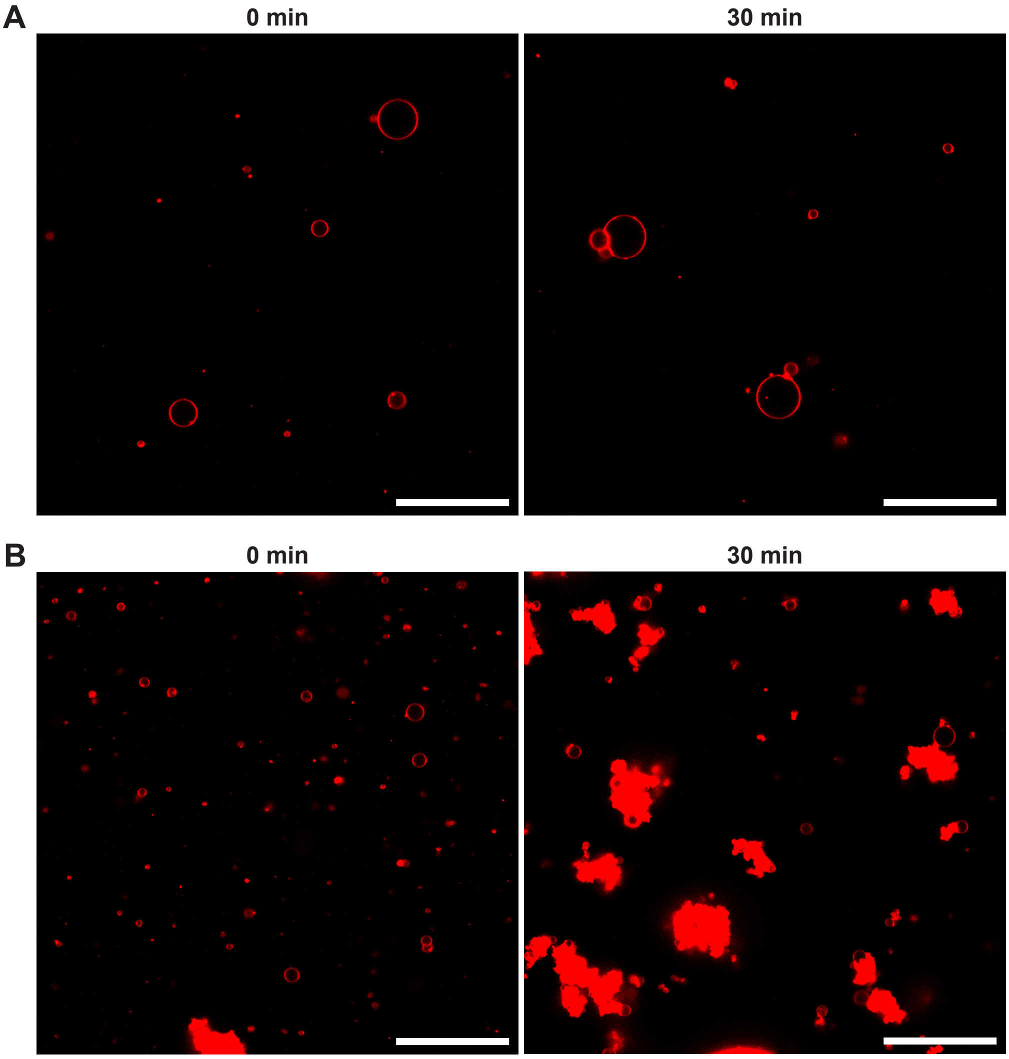

Figure S6. GUV and SecGUV aggregation in the translocation reaction mix. (A) Regular GUVs in the 0 and translocation reaction mix at 37°C and after 30 minutes incubation. Lipid composition DOPC:DOPG:DOPE 40:30:30 with 0.1 mol% added DOPE-ATTO655 (red channel). (B) SecGUVs in the translocation reaction mix at 37°C after 0 and 30 minutes incubation. After 30 minutes incubation, SecGUV aggregation can be observed. Lipid composition DOPC:DOPG:DOPE 40:30:30 with 0.1 mol% added DOPE-ATTO655 (red channel). Protein:lipid ratio 1:500. Scale bars 50 μm.

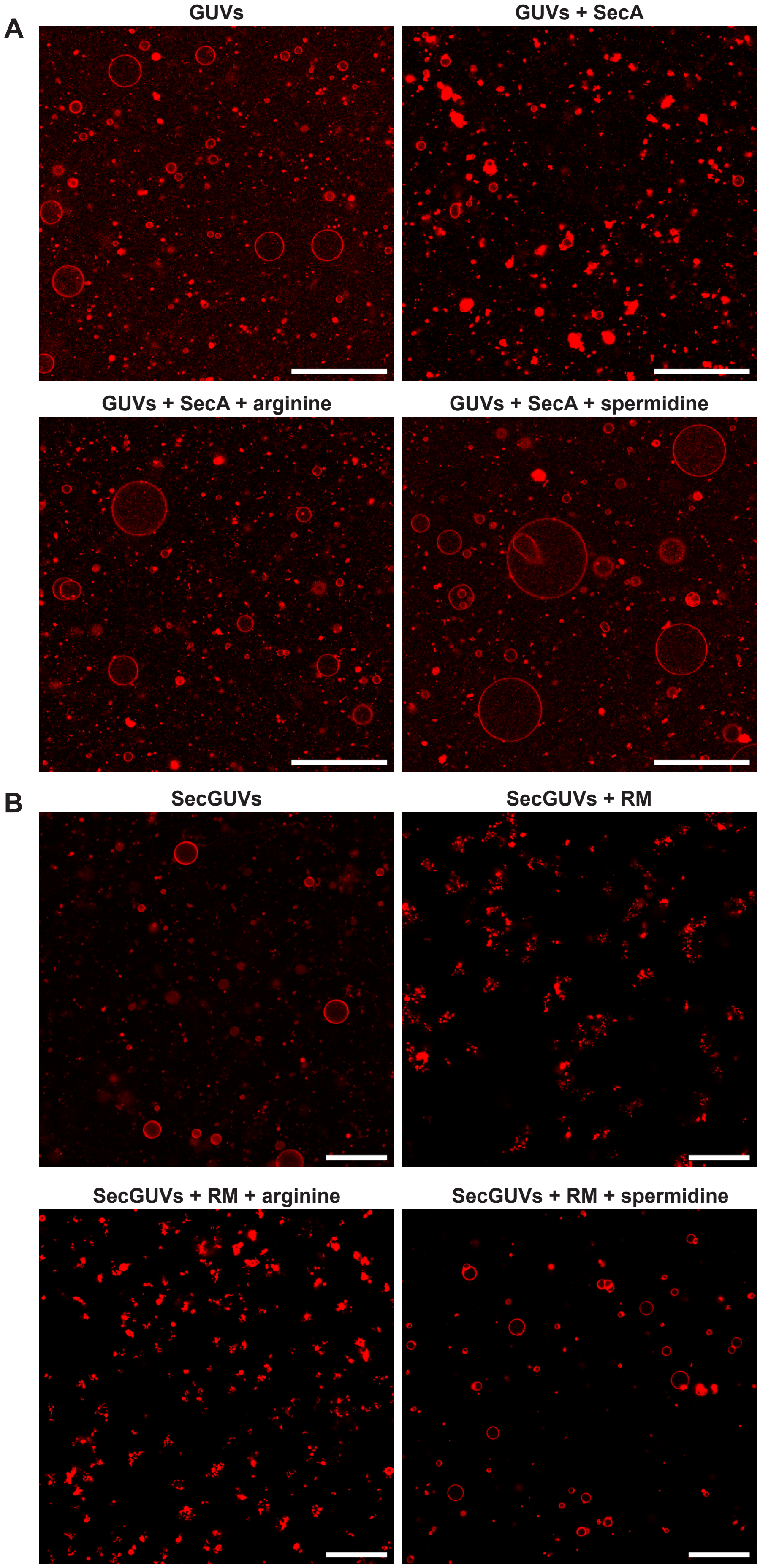

Figure S7. Effect of arginine and spermidine on GUV and SecGUV aggregation. (A) Regular GUVs with composition DOPC:DOPG:DOPE 40:30:30 supplemented with 0.1 mol% DOPE-ATTO655 (red channel). Addition of 250 nM SecA leads to aggregation. Both 50 mM arginine and 10 mM spermidine significantly reduce GUV aggregation. Scale bars 50 µm. (B) SecGUVs with composition DOPC:DOPG:DOPE 40:30:30 supplemented with 0.1 mol% DOPE-ATTO655 (red channel) and with a protein:lipid ratio of 1:500. Addition of SecGUVs to the final reaction mixture consisting of 15 µM proOmpA-MF5, 20 mM Tris-HCl pH 7.4, 0 mM KCl, 10 mM DTT, 0.1 mg.ml BSA, 50 µg/ml creatine kinase, 10 mM phosphocreatine, 250 nM SecA, 500 nM SecB, 2 mM ATP leads to complete aggregation. Addition of 50 mM arginine does not seem to reduce aggregation, but addition of 10 mM spermidine does. Scale bars 50 µm.

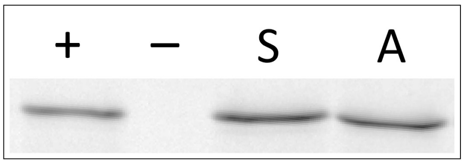

Figure S8. Effect of arginine and spermidine on proOmpA translocation. Translocation assay on SecSUVs: regular translocation reaction (+); negative control, no ATP (-); regular translocation reaction with addition of 10 mM spermidine (S); regular translocation reaction with addition of 50 mM arginine (A). Addition of arginine and spermidine has no influence on translocation activity.

3. Confocal translocation assay

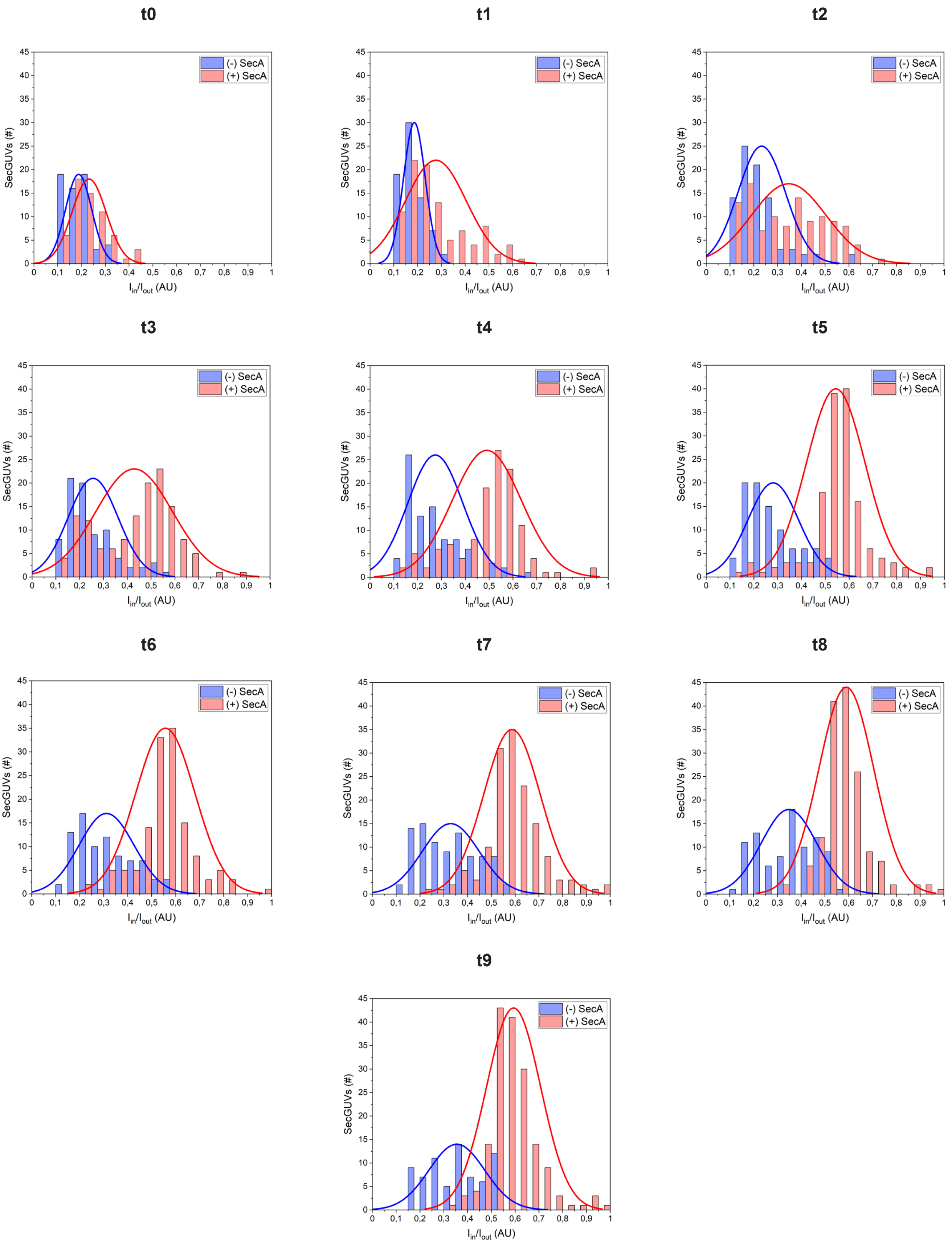

Figure S9. Overview of histograms of the intensity ratios I(in)/I(out) for the positive and negative samples at each time point. Time between time point is equal to 10 minutes.

Timepoint	Count (#)	Average I_{in}/I_{out} (AU)	Sample error I_{in}/I_{out} (AU)	Diameter (µm)	Sample error diameter (µm)
Negative sample					
t0	61	0,188	0,055	11,18	3,35
t1	72	0,186	0,046	12,30	2,98
t2	86	0,233	0,100	12,62	2,96
t3	80	0,256	0,105	12,63	3,39
t4	86	0,274	0,116	12,61	3,01
t5	91	0,281	0,106	12,22	3,21
t6	82	0,312	0,115	12,44	2,96
t7	80	0,332	0,118	12,61	3,33
t8	68	0,351	0,118	12,97	3,40
t9	71	0,355	0,117	12,14	3,47
Positive sample					
t0	60	0,232	0,072	10,81	2,79
t1	98	0,277	0,128	12,13	2,93
t2	110	0,347	0,157	12,45	2,81
t3	135	0,429	0,161	12,51	2,79
t4	122	0,490	0,145	12,31	2,93
t5	146	0,544	0,123	11,87	3,15
t6	136	0,557	0,124	12,41	2,91
t7	142	0,584	0,118	11,65	2,76
t8	157	0,590	0,115	11,21	2,65
t9	171	0,594	0,114	11,08	2,62

Figure S10. Overview of SecGUV counts, average intensities, and sizes per sample per time point. Time between time points is equal to 10 minutes.

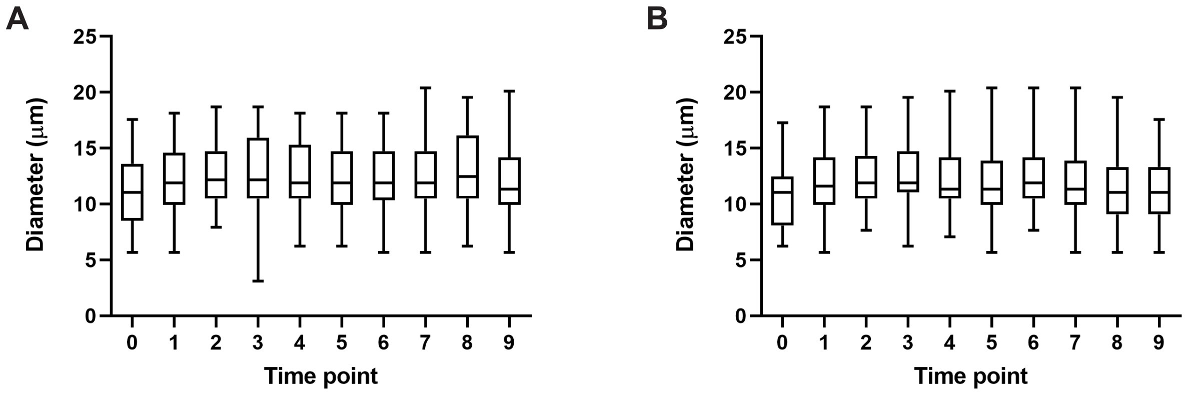

Figure S11. Graphic representation of size distributions of SecGUVs per time point in each sample. (A) Negative sample. (B) Positive sample. Samples showed similar size distributions at each time point.

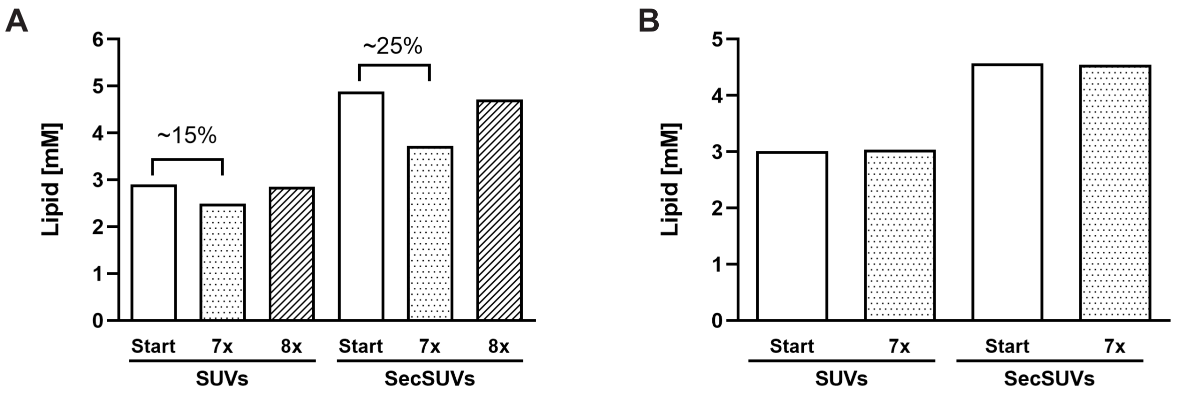

Figure S12. Effect of number of extrusion passes on lipid loss. (A) Loss of lipids due to an uneven number of extrusions of regular SUVs and SecSUVs using a 50 nm pore size polycarobnate filter, compared to an even number of extrusions. For an even number of extrusions, SUVs and SecSUVs are collected from the same syringe in which these samples started. For an uneven number of extrusions, SUVs and SecSUVs are collected from the syringe opposite the starting syringe. In the latter case, lipid material is thought to stay behind on the filter due to the small pore size, leading to a loss of lipid material between 15 and 25%. (B) Loss of lipids due to an uneven number of extrusions using a 100 nm pore size polycarobnate filter. Compared to a 50 nm pore size filter, no lipids are lost for this condition. Lipid concentrations were determined using LC-MS, as described in the Materials and Methods.

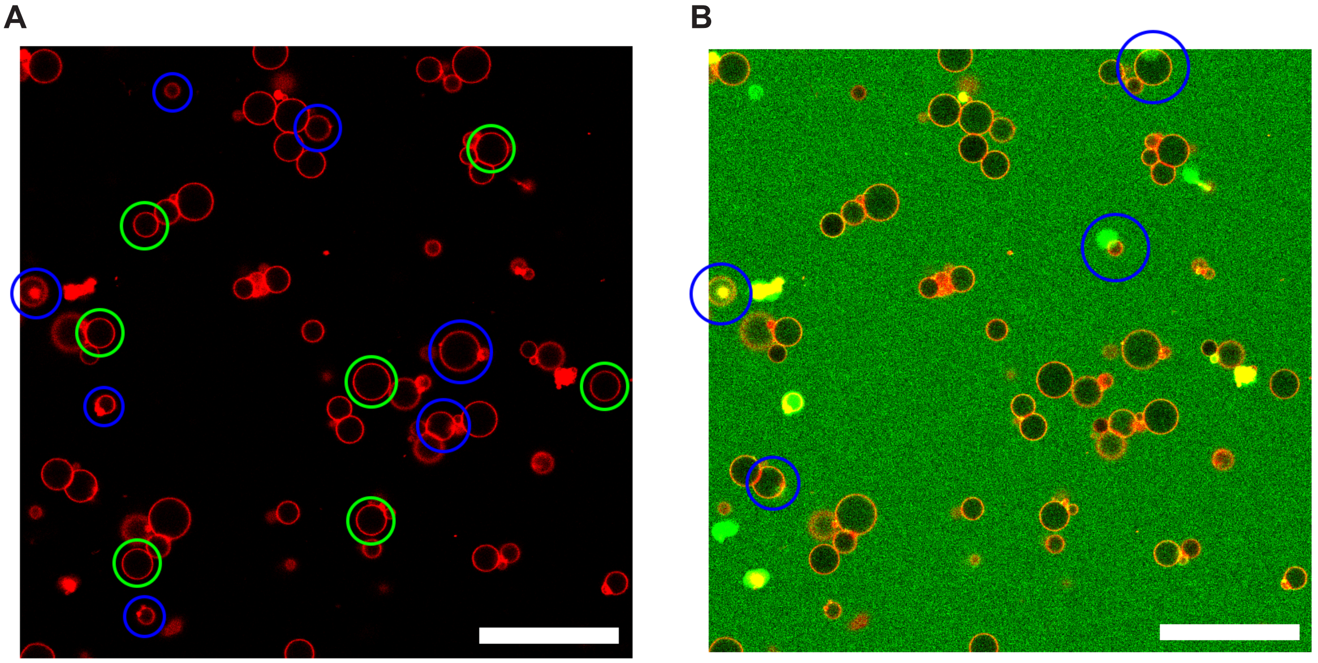

Figure S13. Selection criteria for SecGUV intensity ratio measurements. (A) SecGUVs in red channel. Green circles indicate SecGUVs that have been taken along in the analysis. These SecGUVs have a clear membrane and are in focus. Blue circles indicate the SecGUVs that have been excluded, because these are: not in focus, aggregated, deflated, or otherwise malformed. (B) Overlay of red channel (DOPE-ATTO655) and green channel (proOmpA-5-MF). Blue circles indicate SecGUVs that may be in focus, but are excluded due to proOmpA-5-MF aggregates overlapping with the SecGUV interior. Scale bars 50 µm.

4. SecGUV detection and size determination

Supporting Information S14. ImageJ (Fiji) script used to identify clean and in focus SecGUVs as per the criteria specified in Figure S13. Briefly, the script is based on Weka segmentation to identify SecGUVs, Hough Circle Transform to clean up the probability maps produced by Weka segmentation, and standard ImageJ functions in order to determine the sizes and intensities of the identified SecGUVs. Weka segmentation was trained using a series of images where suitable SecGUVs as well as aggregated, deflated, out-of-focus, and malformed SecGUVs were manually indicated.The eventual intensity measurement of each suitable SecGUV was determined manually.

Script:

```
// this script works on an active 2-channel TIFF (green-red) image, which has been opened in FIJI and is the active window
// the second channel is a neutral marker for GUVs, the first channel contains signals to be measured inside/outside the GUVs

// Weka should be upgraded with the “ImageScience” library
// The required Hough Circle Transform can be obtained by adding the “UCB Vision Sciences” update site in FIJI
// Weka currently (2020.06.23) has a bug when used in a macro; The solution implemented is to put a small (1s) delay after calling Weka

// Replace the file path of the classifier.model to be used by Weka in this macro in the following line:

classifier_address = “ model_x”

// the actual macro starts here

// preparing the image for analysis

        n = getTitle();
        Stack.setChannel(1);
        setMinAndMax(0, 65535);
        Stack.setChannel(2);
        setMinAndMax(0,65535);
        run(“Duplicate...”, “title=GREEN duplicate channels=1”);
        selectWindow(n);
        run(“Duplicate...”, “title=RED duplicate channels=2”);
        run(“Set Measurements...”, “mean redirect=None decimal=6”);

// obtaining green background by excluding objects in both channels

        roiManager(“reset”);

        // pick up green objects

        selectWindow(“GREEN”);
        run(“Duplicate...”, “title=GREEN2”);
        run(“Enhance Contrast”, “saturated=0”);
        run(“8-bit”);
        run(“Gaussian Blur...”, “sigma=1”);
        run(“Manual Threshold...”, “min=100 max=255”);
        run(“Convert to Mask”, “background=Dark black”);
        run(“Dilate”, “slice”);
        run(“Dilate”, “slice”);
        run(“Create Selection”);
        roiManager(“Add”);
        close(“GREEN2”);

        // pick up red objects

        selectWindow(“RED”);
        run(“Duplicate...”, “title=RED2”);
        run(“Enhance Contrast”, “saturated=0”);
        run(“8-bit”);
        run(“Gaussian Blur...”, “sigma=1”);
        setAutoThreshold(“Huang dark”);
        run(“Convert to Mask”, “background=Dark black”);
        run(“Dilate”, “slice”);
        run(“Dilate”, “slice”);
        run(“Create Selection”);
        roiManager(“Add”);
        close(“RED2”);

        // make combined selection of objects

        roiManager(“Deselect”);
        roiManager(“Combine”);
        run(“Create Mask”);
```

```
		roiManager("reset");
		run("Analyze Particles...", "include add");

		// measure value of remaining area in green

		selectWindow("GREEN");
		roiManager("Combine");
		run("Make Inverse");
		run("Measure");
		selectWindow("Results");
		IJ.renameResults("Mean green background intensity of: " + n);

// obtaining a GUV-membrane probability map using Weka

		selectWindow("RED");
		run("Select None");
		run("Duplicate...", "title=RED3");
		run("Trainable Weka Segmentation");
		selectWindow("Trainable Weka Segmentation v3.3.2");
		wait(1000);
		call("trainableSegmentation.Weka_Segmentation.loadClassifier", classifier_address);
		selectWindow("Trainable Weka Segmentation v3.3.2");
		call("trainableSegmentation.Weka_Segmentation.getProbability");
		close("RED3");
		selectWindow("Probability maps");
		Stack.setChannel(2);
		run("Delete Slice");

// thresholding probability map to get binary image

		setAutoThreshold("Huang dark");
		run("Convert to Mask");
		close("Threshold");
		close("Trainable Weka Segmentation v3.3.2");

// obtaining circles out of the binary image using hough

		selectWindow("Probability maps");
		run("Set Measurements...", "  redirect=None decimal=6");
run("Hough Circle Transform","minRadius=10, maxRadius=75, inc=1, minCircles=1, maxCircles=300, threshold=0.75, resolution=1000, ratio=1.0, bandwidth=10, local_radius=10,  reduce results_table");

		// waiting for hough to finish

			while (nResults == 0) {
				wait(500);
				}

// adding resulting circles to the roi manager

		roiManager("reset");

		for (i = 0; i < nResults(); i++) {
		    p = getResult("X (microns)", i);
		    q = getResult("Y (microns)", i);
		    r = getResult("Radius (microns)", i);
		    run("Specify...", "width=" + 2*r + " height=" + 2*r + " x=" + (p+0.30) + " y=" + (q+0.25) + " oval centered scaled");
		    roiManager("add");
			}

		close("Results");

// deleting rois that touch the image border, or if it contains crap in the green channel

		selectWindow("GREEN");
		run("Duplicate...", "title=GREEN3");
		run("Gaussian Blur...", "sigma=1");

		for (i = roiManager("count") -1; i >= 0; i--) {
			roiManager("select", i);
			getSelectionBounds(x, y, w, h);

			// touches border?

			if (x<=0 || y<=0 || x+w>=getWidth || y+h>=getHeight) {
			roiManager("delete");
			}

			// contains green crap?
```

```
                run("Enlarge...", "enlarge=-2 pixel");
                if ((getValue("Max")) > (25 * (getValue("Mean")))) {
                roiManager("delete");
                }

        }

        close("GREEN3");

// preparing scaled images for presentation

        selectWindow(n);
        Stack.setChannel(1);
        run("Enhance Contrast", "saturated=0.1");
        Stack.setChannel(2);
        run("Enhance Contrast", "saturated=0.2");
        Stack.setDisplayMode("composite");
        rename("GUVS_detected");
        roiManager("Deselect");
        run("Select None");
        roiManager("Combine");

        selectWindow("Probability maps");
        rename("Thresholded_Probability");

// measuring desired properties for each remaining circle

        selectWindow("GREEN");
        run("Set Measurements...", "mean min centroid feret's redirect=None decimal=3");
        run("Set Scale...", "distance=0 known=0 pixel=0 unit=[]");
        roiManager("Deselect");
        roiManager("Measure");

                // stop macro when there are no detected circles

                if (nResults == 0) {
                print("No suitable GUVs detected");
                exit
                }

// deleting clutter columns

        IJ.renameResults("GUV results: Green intensity mean/min/max, Centroid x/y (pixels), Diameter (pixels) of: " +n);
        Table.deleteColumn("FeretX");
        Table.deleteColumn("FeretY");
        Table.deleteColumn("FeretAngle");
        Table.deleteColumn("MinFeret");
        Table.renameColumn("Feret", "Diameter");

// cleaning up open images

        close("GREEN");
        close("RED");
        close("Mask");
        run("Tile");
```
